# Unveiling Metabolic Subtypes in Endometrial Cancer Cell Lines: Insights from Metabolomic Analysis Under Standard and Stress Conditions

**DOI:** 10.3390/ijms26199573

**Published:** 2025-09-30

**Authors:** Lana McCaslin, Simon Lagies, Daniel A. Mohl, Dietmar A. Plattner, Markus Jäger, Claudia Nöthling, Matthias C. Huber, Ingolf Juhasz-Böss, Bernd Kammerer, Clara Backhaus

**Affiliations:** 1 Core Competence Metabolomics, Hilde-Mangold-Haus, University of Freiburg, 79104 Freiburg, Germany; 2 Department of Obstetrics & Gynecology, Medical Center and Faculty of Medicine—University of Freiburg, Hugstetter Str. 55, 79106 Freiburg, Germany; 3 Institute of Organic Chemistry, University of Freiburg, 79104 Freiburg, Germany; 4 Signaling Research Centre BIOSS, University of Freiburg, 79104 Freiburg, Germany; 5 Spemann Graduate School of Biology and Medicine (SGBM), University of Freiburg, 79104 Freiburg, Germany

**Keywords:** endometrial cancer, metabolic profiling, metabolic subtypes, mass spectrometry, GC/MS, LC/MS, hypoxia, lactic acidosis, cancer metabolism

## Abstract

Endometrial carcinoma (EC) is the most common malignancy of the female reproductive tract, with increasing incidence driven by aging populations and obesity. While molecular classification has improved diagnostic precision, the identification of clinically relevant metabolic biomarkers remains incomplete, and targeted therapies are not yet standardized. In this study, we investigated metabolic alterations in four EC cell lines (AN3-CA, EFE-184, HEC-1B and MFE-296) compared to non-malignant controls under normoxic and stress conditions (hypoxia and lactic acidosis) to identify metabolomic differences with potential clinical relevance. Untargeted gas chromatography–mass spectrometry (GC/MS) and targeted liquid chromatography–mass spectrometry (LC/MS) profiling revealed two distinct metabolic subtypes of EC. Cells of metabolic subtype 1 (AN3-CA and EFE-184) exhibited high biosynthetic and energy demands, enhanced cholesterol and hexosyl-ceramides synthesis and increased RNA stability, consistent with classical cancer-associated metabolic reprogramming. Cells of metabolic subtype 2 (HEC-1B and MFE-296) displayed a phospholipid-dominant metabolic profile and greater hypoxia tolerance, suggesting enhanced tumor aggressiveness and metastatic potential. Key metabolic findings were validated via real-time quantitative PCR. This study identifies and characterizes distinct metabolic subtypes of EC within the investigated cancer cell lines, thereby contributing to a better understanding of tumor heterogeneity. The results provide a basis for potential diagnostic differentiation based on specific metabolic profiles and may support the identification of novel therapeutic targets. Further validation in three-dimensional culture models and ultimately patient-derived samples is required to assess clinical relevance and integration with current molecular classifications.

## 1. Introduction

Endometrial cancer (EC)—a malignancy of the uterine lining [1]—is the most prevalent gynecological cancer in developed countries [2]. Global incidence has been rising, reaching over 400,000 new cases in 2020, with the highest burden being in North America and Europe [1]. This trend is largely attributed to aging populations and increasing rates of obesity and metabolic disorders, both of which are major risk factors for EC [2]. Obesity contributes to EC primarily by increasing estrogen levels [3]. Estrogen stimulates endometrial proliferation, especially in the absence of opposing progesterone [4]. Factors that elevate estrogen exposure—whether endogenous (like early menarche [5], late menopause [4] or anovulation [5]) or exogenous (such as hormone replacement therapy without progesterone [4])—increase EC risk. Additional risk factors include age, diabetes mellitus, use of tamoxifen and metabolic syndrome [4]. The balance between estrogen and progesterone is crucial, as progesterone inhibits endometrial growth and counteracts estrogenic effects via progesterone receptors (PRs) [6]. Clinically, abnormal uterine bleeding is the most common presenting symptom of EC, though some early-stage cases may be asymptomatic [4]. Advanced disease may present with pelvic pain, uterine enlargement [7] and systemic symptoms such as constipation or fatigue [4]. Diagnosis typically begins with a transvaginal ultrasound [7], followed by an endometrial biopsy for histological confirmation [8]. Advanced imaging techniques like Magnetic Resonance Imaging (MRI) or Computer Tomography (CT) are used to assess disease spread [9]. Treatment is primarily surgical—usually a total hysterectomy with bilateral salpingo-oophorectomy [7]. Postoperative adjuvant therapy is tailored to the cancer’s stage, grade, histological subtype and molecular profile [10]. Radiation therapy (either vaginal brachytherapy or external beam radiation [7]), chemotherapy (typically carboplatin and paclitaxel [10]), hormone therapy and immunotherapy are employed based on clinical indications [4].

A significant advance in EC management is the molecular classification system introduced by The Cancer Genome Atlas (TCGA) [8]. It categorizes EC into four subtypes [4,11]:

1. POLE-mutated: Characterized by ultramutation and good prognosis.
2. Mismatch repair deficient (MMRd): Loss of MMR proteins, intermediate prognosis and responsive to immunotherapy.
3. p53-abnormal (p53abn): TP53 mutations or abnormal p53 expression associated with poor outcomes.
4. No specific molecular profile (NSMP): A molecular heterogeneous group that does not fit into the other three categories with intermediate prognosis.

This classification is determined through immunohistochemistry (IHC) for mismatch repair proteins and p53, and sequencing for POLE mutations. It is now an integral part of diagnostic and therapeutic decision-making [4].

EC cells, like many other malignancies, undergo metabolic reprogramming to support uncontrolled proliferation, growth, resistance to apoptosis and adaptation to the tumor microenvironment (TME) [12]. One of the best documented features of cancerous metabolic reprogramming is the Warburg effect, first described in the 1920s [13]. In this phenomenon, cancer cells preferentially convert glucose to lactate even when oxygen is available, thereby favoring glycolysis over oxidative phosphorylation [14,15]. A key consequence of the Warburg effect is lactic acidosis (LA)—a buildup of lactate and protons that significantly lowers the extracellular pH in tumors [16]. This acidic environment, typically with a pH of 6.0 to 6.5, compared to the normal 7.5, significantly influences tumor physiology [17]. LA promotes angiogenesis, enabling tumors to form new blood vessels [18], and lactate itself serves as an energy source [19]. More importantly, acidic pH impairs immune surveillance, particularly inhibiting T-cell function [20], making lactic acid a potent immunosuppressive metabolite that supports immune evasion and resistance to therapy [21]. A molecular explanation for the Warburg effect is the overexpression of the glycolytic enzyme lactate dehydrogenase A (LDHA), a target of the oncogene MYC, further linking glycolysis to oncogenic signaling [15]. Hypoxia, or low oxygen availability, is another defining feature of the TME and contributes substantially to tumor aggressiveness and poor prognosis [22,23]. As rapidly proliferating cancer cells outstrip their oxygen supply, hypoxia becomes more pronounced, exacerbated by defective tumor vasculature [23,24]. Hypoxia-inducible factors (HIFs), especially HIF-1α, mediate the cellular response to hypoxia by promoting glycolysis and repressing mitochondrial respiration [25]. This not only enhances glucose uptake and lactate production but also contributes to acidification and metabolic shifts similar to those seen in the Warburg effect [25].

The field of metabolomics in EC is still developing. Current research focuses on identifying informative metabolic biomarkers in patient serum and plasma that differ significantly between patients and healthy controls [26]. In serum, certain metabolites like 1-methyladenosine, acylcarnitines and ceramides have been identified [27]. Most metabolites identified in previous metabolomic research on EC are associated with lipid, glucose and amino acid metabolism. Building on these findings, our study presents a comprehensive metabolomic approach combining the analysis of modified nucleosides (mNs), lipids, and intra- and extracellular metabolites in EC.

Using high-performance liquid chromatography coupled with triple quadrupole mass spectrometry (HPLC-QqQ-MS), we conducted a semi-quantitative analysis of 62 mNs, representing, to our knowledge, the most comprehensive panel of mNs studied to date in the context of EC. In addition, targeted lipidomic analyses were performed to investigate the metabolic interplay between lipid dysregulation and endometrial carcinogenesis. Given the established role of obesity as a major EC risk factor [2], alterations in lipid metabolism may provide mechanistic insights into disease development and progression. To further elucidate metabolic reprogramming at the cellular level, gas chromatography (GC)-based metabolite profiling was conducted, enabling a detailed overview of central carbon metabolism and other intracellular metabolic pathways.

Together, our integrative metabolomic approach provides new insights into the metabolic landscape of EC across different biological matrices and levels of biological organization. These findings contribute to a deeper understanding of EC pathophysiology and may support the development of novel metabolic biomarkers or therapeutic targets.

### Aim of Study

The aim of this study was to assess the metabolic profile of EC by identifying distinct metabolic signatures in a panel of EC cell lines under both standard and tumor-relevant stress conditions. Using a multi-cell line model comprising four well-established EC cell lines and non-malignant controls (see Table 1), we investigated intra- and extracellular metabolite profiles, including amino acids, lipids, sugars, and nucleosides via untargeted gas chromatography–mass spectrometry (GC/MS) and targeted high-performance liquid chromatography–triple quadrupole mass spectrometry (HPLC-QqQ-MS).

In addition to metabolic profiling at basal growth conditions, we evaluated the effects of two key tumor microenvironmental stressors—hypoxia and lactic acidosis—on EC cell metabolism. Furthermore, selected findings were validated at the transcript level through quantitative real-time PCR to assess the expression of metabolic enzymes.

This study aims to provide novel insights into the metabolic equipment and heterogeneity of EC and to identify candidate metabolic biomarkers and fingerprints associated with tumor progression and stress adaptation.

## 2. Results and Discussion

### 2.1. Comprehensive Exo- and Endo-Metabolomic Analysis of Endometrial Cell Lines and Their Culture Media

**Figure 1:**
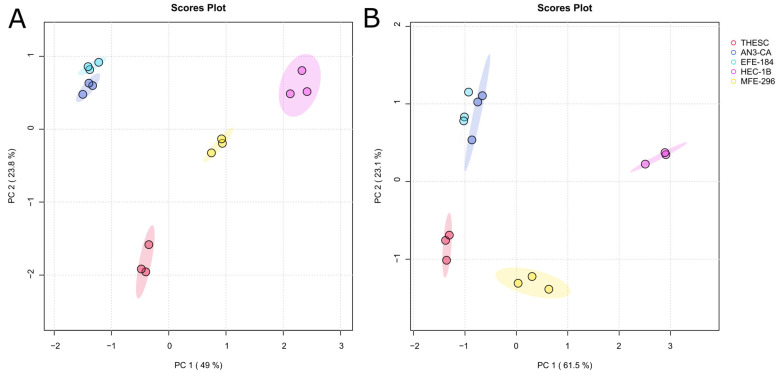
Principal component analysis (PCA) of intra- (**A**) and extracellular (**B**) nucleosides. PCA indicates differences between samples and groups. Shaded areas represent 95% confidence ellipses. Percentage of variance explained by each principal component reaching 49.0% for PC1 and 23.8% for PC2 in (**A**), as well as 61.5% for PC 1 and 23.1% for PC2 in (**B**). Individual samples of a group are indicated by colored dots within the confidence ellipse. Each cell line, such as AN3-CA, EFE-184, HEC-1B, MFE-296 and THESC, was analyzed in biological triplicate (*N* = 3 for each cell line).

Cultured cancer cell lines and their surrounding culture medium offer a simplified in vitro model to explore tumor-associated metabolic processes. While the cells themselves reflect the intracellular metabolic activity, which represents the endo-metabolome, the culture medium provides supplementary information on secreted or exchanged metabolites, reflecting aspects of the TME and the exo-metabolome. Figure 1 shows the PCA (principal component analysis) score plots of endo- (Figure 1A) and exo-nucleosides (Figure 1B) analyzed in cells and CCM of EC cell lines and a non-malignant control cell line. Both score plots reveal three distinct groups: two groups of EC cell lines (AN3-CA and EFE-184, as well as HEC-1B and MFE-296) and one group of control cell lines (Telomerase-immortalized Human Endometrial Stromal cells (THESCs)). This demonstrates that intracellular and extracellular nucleoside profiles of EC cell lines cluster into two clearly distinguishable groups, each displaying high internal consistency.

**Figure 2:**
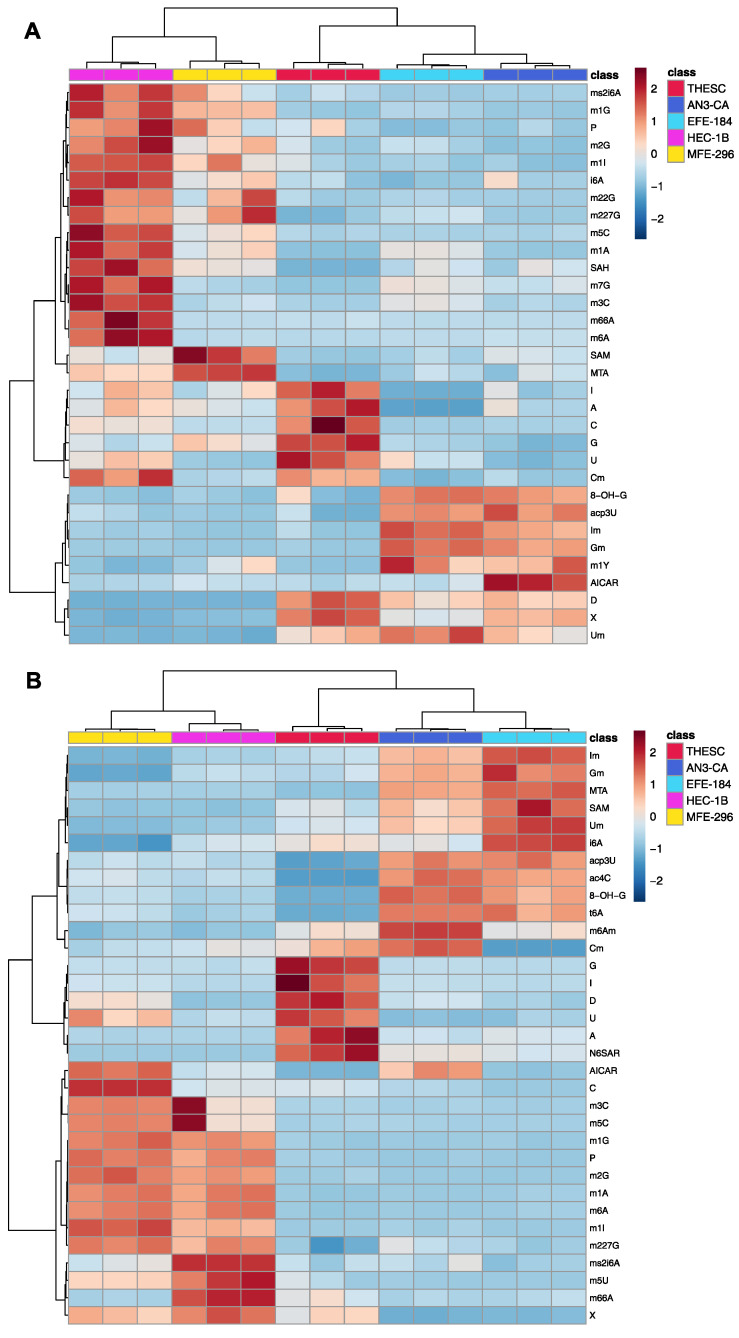
Clustered heat map representation of intra- (**A**) and extracellular (**B**) nucleoside analysis. The color scale on the right indicates the relative concentrations in the form of range-scaled z-scores, where red hues represent higher relative concentrations and blue hues represent lower relative concentrations. Only significantly altered nucleosides are shown in both heat maps (one-way ANOVA, *p* < 0.05 after FDR). Each cell line was analyzed in biological triplicate (*N* = 3). A list of all abbreviated nucleosides is presented in Supplementary Table S1.

Both nucleoside analyses are also displayed in the clustered heat maps in Figure 2. The three groups present in the PCA described above are reflected in the endo-metabolomic nucleoside cluster analysis as well (Figure 2A). Non-malignant cells (THESCs) display the highest levels of unmodified nucleosides such as adenosine (A), cytidine (C), guanosine (G) and uridine (U). In contrast, modified nucleosides are found in higher amounts in EC cells. The increased levels of modified nucleosides observed in EC cells may reflect enhanced RNA turnover and disturbed RNA homeostasis, both of which are commonly associated with cancer [30]. AN3-CA and EFE-184 cells form a cluster characterized by high levels of ribose-methylated nucleosides, including 2′-O-methylguanosine (Gm), 2′-O-methylinosine (Im) and 2′-O-methyluridine (Um), which have been associated with enhanced RNA stability and mechanisms of immune evasion [31]. The presence of 8-hydroxyguanosine (8-OH-G) indicates increased oxidative stress and may be linked to increased RNA oxidation in cancer cells [32]. High levels of 5-aminoimidazole-4-carboxamide ribonucleotide (AICAR), as an intermediate in the synthesis of purines, indicate enhanced purine biosynthesis and turnover [33]. The HEC-1B and MFE-296 cluster contains mainly methylated adenosines like 1-methyladenosine (m1A), N6-methyladenosine (m6A) and N6N6-dimethyladenosine (m66A), as well as methylated guanosines like 1-methylguanosine (m1G), N2-methylguanosine (m2G), N2N2-dimethylguanosine (m22G), N2N2,7-trimethylguanosine (m227G) and 7-methylguanosine (m7G). Although high m6A levels are unexpected—given that approximately 70% of EC exhibit decreased m6A methylation [34], high m1A levels align with previous findings in patient serum [27], reinforcing its potential as a diagnostic biomarker. Notably, aberrant expression of m7G methyltransferases is frequently observed in various cancers [35] and has been implicated in promoting malignant phenotypes, including increased invasiveness, metastatic potential, resistance to apoptosis, immune evasion and metabolic reprogramming [36]. Consequently, m7G-related pathways are emerging as promising therapeutic targets.

Comparable clustering of EC cells as before (intracellular) can be seen in the extracellular nucleoside analysis (Figure 2B). The nucleosides, which were elevated in the endo-metabolic analysis, were also elevated in the exo-metabolic clustered heat map analysis: control cells show an accumulation of unmodified nucleosides, whereas AN3-CA and EFE-184 cells exhibit increased levels of ribose methylated nucleosides, and HEC-1B and MFE-296 cells are characterized by elevated levels of methylated adenosines and guanosines.

Since the cluster analysis for intracellular and extracellular nucleosides shows two distinct clusterings of two cancer cell lines, we concluded a metabolic subtyping classification system: EC subtype 1 (AN3-CA + EFE-184) and EC subtype 2 (HEC-1B + MFE-296). This metabolomic cancer classification will be used in subsequent experiments. In the following, the terms subtype 1 and subtype 2 refer to this metabolomic-based subtyping of EC.

All in all, EC subtypes exhibit consistent nucleoside profiles across both endo- and exo-metabolomic analyses, with the same nucleosides being elevated in both compartments. This analysis highlights that the unique nucleoside signatures of EC cell lines can discriminate between EC subtypes. As the nucleoside profile enables molecular characterization of cell lines, it has the potential to contribute to the discovery of new and clinically relevant biomarkers. Consequently, further analyses based on the metabolic EC subtypes were conducted in this study to determine phenotypic correlations and validate the relevance for the early detection and targeted therapy of EC.

### 2.2. Metabolomic Analysis of Endometrial Cancer Subtypes

**Figure 3:**
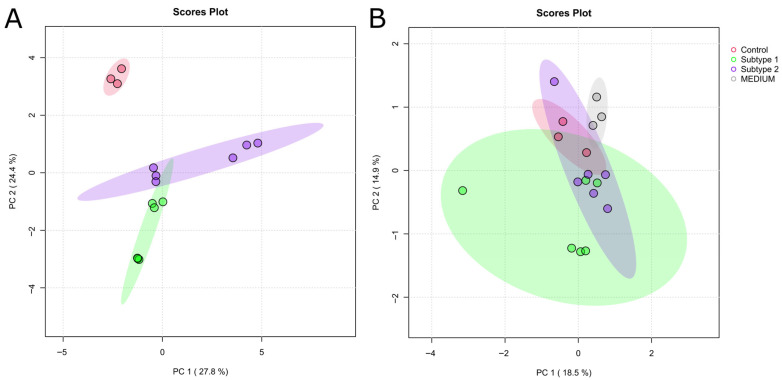
PCA of intra- (**A**) and extracellular (**B**) metabolites in EC subtypes. PCA indicates differences between samples and groups. Shaded areas represent 95% confidence ellipses. Percentage of variance explained by each principal component reaching 27.8% for PC1 and 24.4% for PC2 in (**A**), as well as 18.5% for PC 1 and 14.9% for PC2 in (**B**). Individual samples of a group are indicated by colored dots within the confidence ellipse. Each cell line, such as AN3-CA, EFE-184, HEC-1B, MFE-296 and THESC, and cell-free CCM, was analyzed in biological triplicate (*N* = 3 for CCM and each cell line).

Figure 3 shows the PCA score plots of intracellular (Figure 3A) and extracellular (Figure 3B) metabolites in cells and CCM, respectively, which were analyzed using untargeted metabolic profiling by GC/MS and lipidomics by targeted LC/MS. It displays marked differences between control and EC cells as well as between the cancer cells of subtypes 1 and 2 (Figure 3A) and fewer differences between all groups regarding extracellular metabolites (Figure 3B).

**Figure 4:**
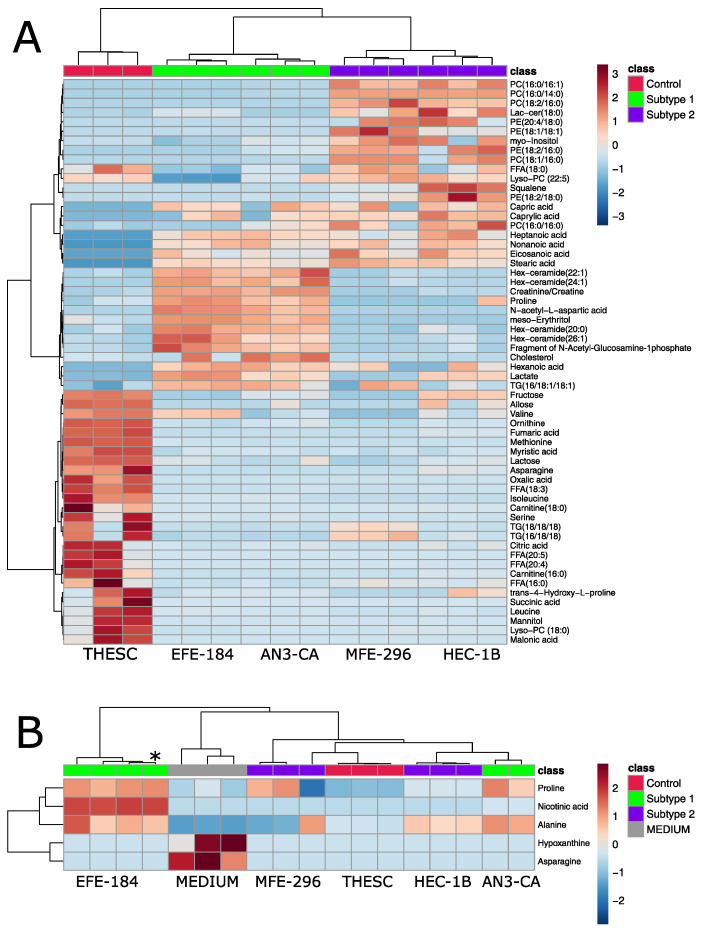
Clustered heat map representation of intra- (**A**) and extracellular (**B**) metabolites in EC subtypes. The color scale on the right indicates the relative concentrations in the form of range-scaled z-scores, where red hues represent higher relative concentrations and blue hues represent lower relative concentrations. Only significantly altered nucleosides are shown in both heat maps (one-way ANOVA, *p* < 0.05 after FDR). Cell-free CCM and each cell line were analyzed in biological triplicate (*N* = 3). Fragment of N-Acetyl-Glucosamine-1-Phosphate could also arise from UDP-N-Acetyl-glucosamine, which cannot be distinguished in GC/MS analysis. A list of all abbreviated lipids is presented in Supplementary Table S2. The first number indicates the number of carbon atoms, and the second number indicates the number of double bonds in the fatty acid. * = replicate of AN3-CA.

Statistical analysis revealed 60 significantly altered intracellular and five significantly altered extracellular metabolites, supporting the fact that there are no major differences in the exo-metabolome between the groups. These significant changes are displayed in clustered heat maps (Figure 4). Among the few altered metabolites detected in the exo-metabolome analysis (Figure 4B), asparagine and hypoxanthine were identified, likely reflecting their role as basic nutrients taken up and consumed by the cells from the medium formulation. Furthermore, exo-metabolome analysis reveals a clustering and partial overlap of cells belonging to EC subtypes 1 and 2 (Figure 3B and Figure 4B).

In contrast, endo-metabolome profiling recapitulates the three distinct groups observed in the nucleoside analysis (Figure 3A and Figure 4A). Control cells and cells of subtypes 1 and 2 are clearly separated from each other, indicating that the EC nucleoside subtyping is also reflected at the level of intracellular metabolites. Control cells have high levels of amino acids, like leucine, methionine, serine and valine, as well as tricarboxylic acid (TCA) cycle products, such as fumaric acid, succinic acid and carnitine. This reflects an active oxidative phosphorylation pathway, cell growth and protein biosynthesis [37]. In contrast, EC cells predominantly relied on aerobic glycolysis, also known as the well-established Warburg effect, for energy production. The metabolic signature of cells of EC subtype 1 reflected elevated energy demands [38], as evidenced by increased levels of lactate and altered lipid metabolism, with significantly elevated cholesterol, hexosyl-ceramides and proline levels, which is important in the remodeling of extracellular matrix, particularly during epithelial–mesenchymal transition (EMT) [39]. Further, higher levels of meso-erythritol suggest the presence of oxidative stress [40], consistent with cancer metabolic reprogramming. These changes are hallmarks of malignant and proliferative phenotypes. The metabolic signature of cells of EC subtype 2 emphasized membrane lipid metabolism and remodeling, suggesting active proliferation [41]. High levels of squalene and phospholipids (phosphatidylcholines (PCs), phosphatidylethanolamines (PEs)) indicated upregulated lipid biosynthesis, likely supporting rapid membrane formation in proliferating cells [41]. The presence of ceramides and lactosylceramides suggested increased activation of the sphingolipid pathway, which is common in tumors [42].

### 2.3. Validation of Metabolomic Endometrial Cancer Subtypes Using qPCR

**Figure 5:**
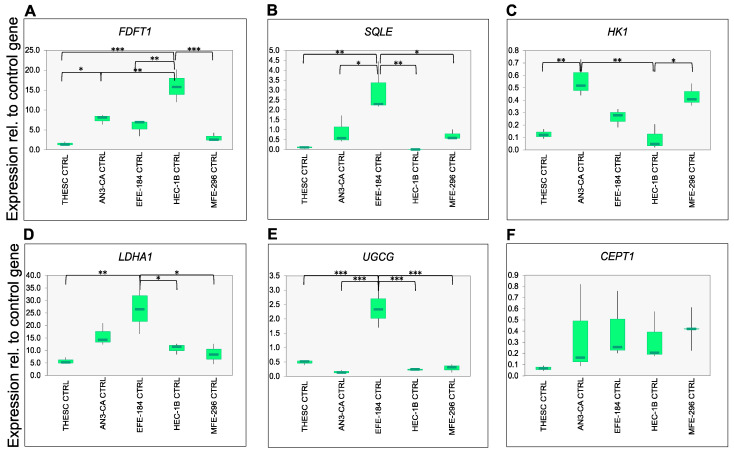
RNA expression of metabolic enzymes in EC cells. (**A**–**F**) RNA expression of selected metabolic enzymes was analyzed for farnesyl-diphosphate farnesyltransferase (**A**), squalene epoxidase (**B**), hexokinase-1 (**C**), lactate dehydrogenase A 1 (**D**), glucosylceramide synthase (**E**), choline phosphotransferase (**F**) and aldehyde dehydrogenase 1 family member A1. Expression of ER membrane protein complex subunit 7 (EMC7) served as a control gene, and expressions were calculated as fold change across all samples using the 2^−ΔΔCt^ method. Significances were shown with symbols *: *p* < 0.05, **: *p* < 0.01, ***: *p* < 0.001. Each cell line was analyzed in biological triplicate.

To validate the metabolic signatures identified by MS, we performed qPCR targeting key metabolic enzymes involved in cholesterol biosynthesis, glycolysis, hexosyl-ceramide synthesis and phospholipid metabolism (Figure 5). In the cholesterol biosynthesis pathway, MS data revealed elevated cholesterol levels in subtype 1 cells and increased levels of squalene, a cholesterol precursor [43], in subtype 2 cells (Figure 4A). Farnesyl-diphosphate farnesyltransferase 1 (FDFT1), the enzyme catalyzing the conversion of farnesyl-diphosphate to squalene [44], showed significantly higher expression in HEC-1B cells (subtype 2) (Figure 5A). In contrast, squalene epoxidase (SQLE), the second rate-limiting enzyme in cholesterol biosynthesis, responsible for converting squalene into downstream sterols [45], was significantly upregulated in EFE-184 cells (subtype 1) (Figure 5B). These results are consistent with the MS data and support a subtype-specific regulation of cholesterol biosynthesis.

Further supporting the metabolic distinctions, lactate levels were found to be increased in subtype 1 cells (Figure 4A). To explore this, we analyzed the expression of hexokinase 1 (HK1), the first rate-limiting enzyme in glycolysis that catalyzes the phosphorylation of glucose to glucose-6-phosphate [46], and LDHA, which converts pyruvate to lactate under anaerobic conditions [47]. LDHA expression was significantly higher in EFE-184 cells, while HK1 expression peaked in AN3-CA cells, both belonging to subtype 1 (Figure 5C,D), thereby supporting increased glycolytic activity, as observed in the MS analysis.

Similarly, hexosyl-ceramides were elevated in subtype 1 cells (Figure 4A). These are synthesized from ceramides via UDP-glucose ceramide glucosyltransferase (UGCG) [48]. Consistent with the MS data, UGCG expression was significantly elevated in EFE-184 cells (Figure 5E), supporting the enhanced formation of hexosyl-ceramides in subtype 1.

In contrast, levels of PC and PE were increased in subtype 2 cells, according to MS data (Figure 4A). These phospholipids are synthesized through the CDP-choline and CDP-ethanolamine pathways, respectively, with choline/ethanolamine phosphotransferase 1 (CEPT1) catalyzing the final step [49]. However, qPCR analysis revealed no significant differences in CEPT1 expression between the subtypes (Figure 5F), indicating a discrepancy between transcript and metabolite levels.

We acknowledge that the validation of our results is currently limited to mRNA expression analysis via qPCR. This represents a clear limitation, as transcript levels do not always correlate directly with protein abundance or activity. Future studies should therefore include protein-level validation as well as functional assays to confirm the metabolic differences observed at the transcriptional level. Nevertheless, the dual confirmation of gene expression changes at both the mRNA level and the metabolite level provides a robust indication of biologically relevant alterations in cellular metabolism.

### 2.4. Metabolomic Analysis of Acidosis and Hypoxia-Treated Endometrial Cancer Subtypes

To closely mimic physiological conditions, our EC cell lines were exposed to hypoxia (HX) and lactic acidosis (AC), as both are well-established hallmarks of the TME [50]. This experimental setup aimed to investigate how these cancer-associated stress conditions influence the metabolic phenotype of the distinct EC subtypes and whether they affect nucleoside profiles at the intra- and extracellular level. AC treatment was validated by measuring the lactate concentration in the corresponding CCM. As expected, the box plots (Supplementary Figure S1A–E) show that the lactate concentration in the CCM increased significantly with the application of lactic acid, validating the AC condition. In addition, all cell lines produce lactate under control and hypoxic conditions, as lactate is elevated relative to the unconditioned CCM. Furthermore, all cell lines take up lactate under acidotic conditions as the lactate concentration is lower than in the unconditioned CCM containing lactic acid. In addition, control cells (Supplementary Figure S1A) have the highest amount of lactate in the CCM under acidotic conditions. In comparison, all four cancer cell lines (Supplementary Figure S1B–E) have approximately the same level of lactic acid in the CCM under all conditions. HX treatment was validated by qPCR analysis of hypoxia-inducible factor-1α (HIF-1α), which accumulates in response to cellular HX and is an endogenous HX marker [51]. HIF-1a tends to be upregulated at the RNA level in all HX-treated cells compared to control cells (Supplementary Figure S1F). However, HIF-1α is only significantly elevated in EC subtype 1 cells (AN3-CA and EFE-184).

**Figure 6:**
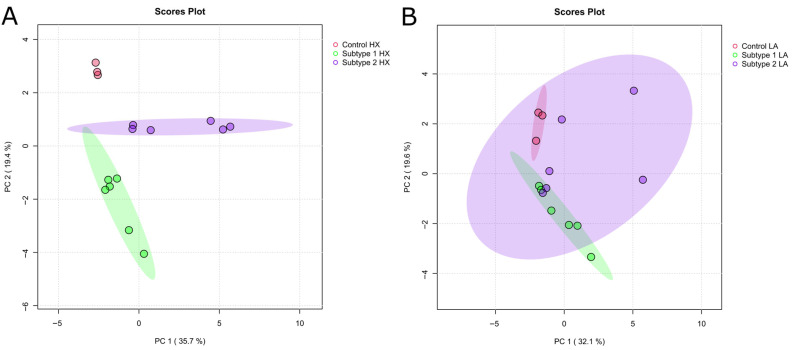
PCA of intracellular metabolites in hypoxia (**A**) and acidosis- (**B**) treated EC subtypes. PCA indicates differences between samples and groups. Shaded areas represent 95% confidence ellipses. Percentage of variance is explained by each principal component, reaching 35.7% for PC1 and 19.4% for PC2 in (**A**), as well as 32.1% for PC 1 and 19.6% for PC2 in (**B**). Fold changes relative to untreated controls were calculated to indicate the direction and magnitude of metabolic alterations. Individual samples of a group are indicated by colored dots within the confidence ellipse. Each cell line, AN3-CA (subtype 1), EFE-184 (subtype 1), HEC-1B (subtype 2), MFE-296 (subtype 2) and THESC (control), was analyzed in biological triplicate (*N* = 3 for each cell line).

Based on the validation of AC and HX treatment, we performed untargeted metabolic profiling by GC/MS and lipidomics by targeted LC/MS analysis of EC cell subtypes. Fold changes relative to untreated controls were calculated to indicate the direction and magnitude of metabolic alterations. Figure 6 shows the PCA score plots of intracellular metabolites from HX-treated (Figure 6A) and LA-treated (Figure 6B) cells. It displays significant differences between HX-treated control and EC subtypes, while AC-treated control and EC subtypes overlap and differ less. Further, EC subtypes overlap in AC treatment but show differentiation in HX-treatment.

**Figure 7:**
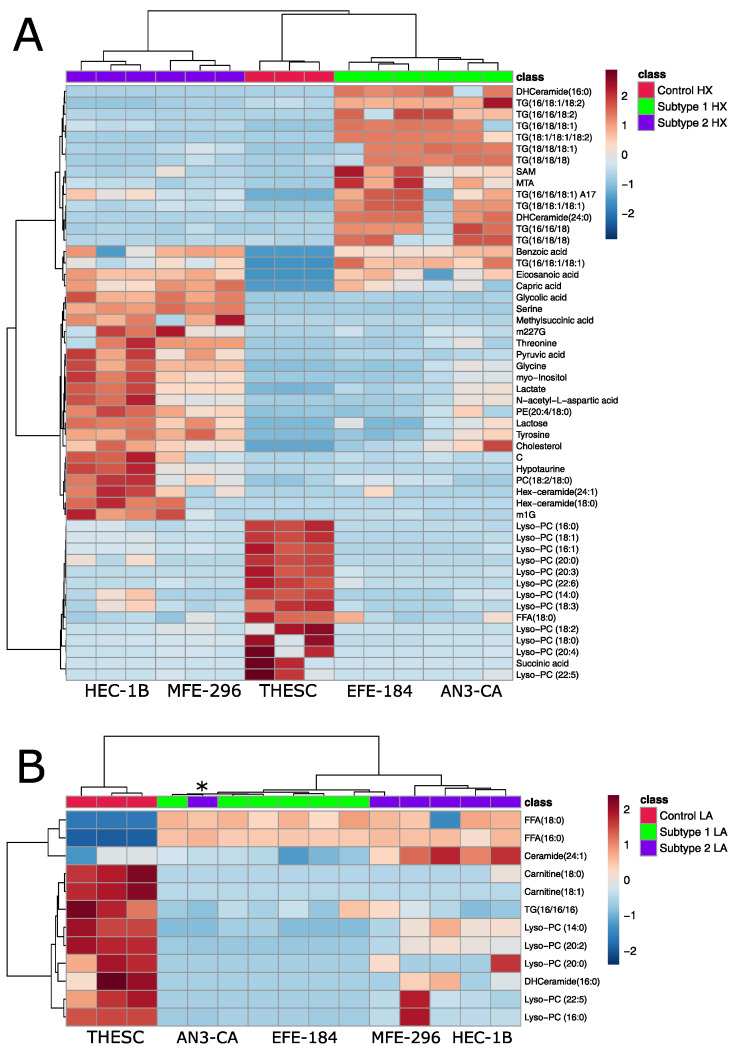
Clustered heat map representation of the intracellular metabolites in hypoxia (**A**) and acidosis- (**B**) treated EC subtypes. Fold changes relative to untreated controls were calculated to indicate the direction and magnitude of metabolic alterations. The color scale on the right indicates the relative concentrations in the form of range-scaled z-scores, where red hues represent higher relative concentrations and blue hues represent lower relative concentrations. Only significantly altered metabolites are shown in both heat maps (one-way ANOVA, *p* < 0.05 after FDR). Each cell line was analyzed in biological triplicate (*N* = 3). Lists of all abbreviated nucleosides and lipids are presented in Supplementary Tables S1 and S2. The first number indicates the number of carbon atoms, and the second number indicates the number of double bonds in the fatty acid. * = replicate of MFE-296.

The metabolism of control and cancer cells adapts differently to HX, as there are 52 significantly altered metabolites (Figure 7A). Under hypoxic conditions, a clustering into three distinct groups (control, subtype 1 and subtype 2 cells) is visible. In contrast, acidotic conditions show a clustering of only two groups: control cells and EC subtypes (Figure 7B). In addition, there are fewer significantly altered metabolites (only 12) than under hypoxic conditions, indicating that control cells and cancer cells are more similar in terms of metabolic adaptation to acidosis.

Under acidotic and hypoxic conditions, control cells exhibited comparable responses characterized by cellular stress, evidenced by elevated levels of lysophosphatidylcholines (lyso-PCs) (Figure 7). This outcome was expected, as both HX and AC are recognized as stress-inducing conditions for non-malignant cells [52]. Lyso-PCs are generated via partial hydrolysis of PCs, typically mediated by phospholipase A2 (PLA2) [53]. During HX, PLA2 activity is upregulated [54], leading to enhanced degradation of membrane phospholipids, compromised membrane integrity and subsequent accumulation of lyso-PCs [52]. Lyso-PCs also function as damage-associated molecular patterns (DAMPs) [55], capable of activating immune cells, inducing pro-inflammatory cytokine release and promoting apoptosis [53].

Similar to control cells, cells of subtype 1 demonstrated signs of cellular stress under HX, indicated by elevated levels of dihydroceramides (DHCeramides). The enzymatic conversion of DHCeramides to ceramides by desaturases involves the introduction of a double bond [56]. Since desaturase activity is oxygen-dependent, HX inhibits this process [57], resulting in DHCeramide accumulation. In addition, cells of subtype 1 showed increased levels of triglycerides (TGs) under HX. The β-oxidation and subsequent mitochondrial oxidative phosphorylation are oxygen-dependent processes [38]. As a result, under HX, the stored TGs cannot be degraded to fatty acids, leading to their accumulation [38]. This is in accordance with other multiple cancer types in which triglycerides are increased under hypoxic conditions [58,59]. These findings indicate that both control and subtype 1 cells experience metabolic dysregulation and disruption of homeostasis under hypoxic stress.

In contrast, cells of subtype 2 appeared to be resistant to HX-induced stress. Amino acids critical for cellular growth and protein biosynthesis—such as glycine, serine, threonine and tyrosine [60] —were significantly increased. Further, elevated levels of lactate and pyruvate suggest enhanced glycolytic activity and energy metabolism [38]. This metabolic profile implies that cells of subtype 2 are not stressed under HX compared to subtype 1 and control cells. This result is further supported by the exclusively significant upregulation of HIF-1α in subtype 1 cells (Supplementary Figure S1F), indicating a more pronounced stress response to the hypoxic environment in this subtype, while subtype 2 cells remain unaffected. Further adaptation to hypoxia can also occur through HIF-independent mechanisms [61]. These include allosteric regulation of glycolytic enzymes [61], increased expression of glucose transporters [62] and enhanced glutaminolysis [63]. Such pathways may contribute to hypoxia tolerance in subtype 2 cells without significant upregulation of HIF-1α.

Unlike the differential response observed under HX, both EC subtypes responded similarly to AC (Figure 7B). Both accumulated free fatty acids (FFAs) under AC, likely due to inhibition of β-oxidation. Acyl-CoA dehydrogenase (ACAD) activity is pH-sensitive: long-chain specific ACADs have optimal activity at a pH of 8.0 [64], while short-chain-specific ACADs have an optimal pH of 7.1 [64]. Acidic conditions impair enzyme function, causing FFA accumulation instead of FFA oxidation.

In general, a notable limitation of this study is the exclusive use of two-dimensional (2D) monolayer cell cultures. While these models are widely used and provide a controlled environment to study cellular metabolism, they lack the structural and microenvironmental complexity inherent to three-dimensional (3D) cultures or patient-derived models. Incorporating such advanced models in future studies will be essential to improve the translational relevance of our metabolic findings.

### 2.5. Establishing Clinically Relevant Context for Novel Metabolic EC Subtypes

In order to comprehensively compare these novel metabolic EC subtypes with the established molecular EC classification from the TCGA, detailed molecular characterization of the used EC cell lines is necessary and remains underrepresented in the current literature. Future studies should include immunocytochemistry (ICC) for mismatch repair (MMR) proteins (e.g., MLH1,PMS2, MSH2 and MSH6 [7]) to identify the MMRd molecular subtype and ICC on p53 to detect the p53abn molecular subtype. Additionally, sequencing of EC cell lines could reveal pathogenic mutations in POLE, TP53, MMR and other genes. Combining metabolic and molecular EC classification could significantly improve diagnostic accuracy, prognostic stratification and guide the development of targeted therapeutic strategies.

## 3. Materials and Methods

### 3.1. Cell Culture

For this study, Telomerase-immortalized Human Endometrial Stromal cells (THESCs) were employed as a non-malignant endometrial control. The endometrial cancer (EC) cell lines AN3-CA, EFE-184, HEC-1B and MFE-296 were used to present malignant phenotypes. The investigated cell lines, presented and described in Table 1, were all cultured as described below. The cells were grown in a Gibco DMEM F12 medium (Life Technologies, Darmstadt, Germany) supplemented with 10% fetal bovine serum (FBS) (Life Technologies, Darmstadt, Germany), 1% Penicillin/Streptomycin (Life Technologies, Darmstadt, Germany) and with 1% HEPES (Sigma-Aldrich/Merck KGaA, Darmstadt, Germany) in a humidified atmosphere containing 5.0% CO_2_ at 37 °C. Cell lines were originally purchased from BioCat (BioCat GmbH, Heidelberg, Germany: THESC) or CLS (CLS Cell Lines Service GmbH, Eppelheim, Germany: AN3-CA, HEC-1B) or DSMZ (Leibniz-Institut DSMZ GmbH, Braunschweig, Germany: EFE-184, MFE-296). The authenticity of cell lines has been regularly confirmed via services from CLS (CLS Cell Lines Service GmbH, Eppelheim, Germany), DSMZ (Leibniz Institute, DSMZ-German Collection of Microorganisms and Cell Cultures GmbH, Braunschweig, Germany) and Multiplexion GmbH (Friedrichshafen, Germany).

Cells were cultured in 75 cm^2^ plastic flasks (Sarstedt AG & Co. KG, Nümbrecht, Germany) and grown at 37 °C/5.0% CO_2_. After reaching confluence, cells were washed with 8 mL 1× PBS (Life Technologies, Darmstadt, Germany) twice and incubated with 3 mL 1× Trypsin/EDTA (Life Technologies, Darmstadt, Germany) at 37 °C/5.0% CO_2_. Trypsinization was stopped by adding 6 mL fresh medium with serum, and cells were reseeded depending on their confluency.

### 3.2. Cell Treatment

For this study, cells were then seeded in 6-well plates (Sarstedt AG & Co. KG, Nümbrecht, Germany) and grown at 37 °C/5.0% CO_2_ for 24 h for attachment. For another 48 h, cells were grown under standard conditions (37 °C/5.0% CO_2_) or subjected to lactic acidosis (cell culture media was supplemented with 2 µL lactic acid; final concentration was 0.2%, pH 6.2) or hypoxia (transfer to hypoxic culture conditions (37 °C, 1% O_2_) in a hypoxic chamber for generation of a hypoxic environment for tissue culture) for subsequent harvesting of cell culture medium (CCM) and cells for analysis of metabolites and nucleosides.

### 3.3. Cell and CCM Harvest

Right before cell harvest, 1.5 mL of cell culture medium was collected, centrifuged to remove cell debris, transferred to a new vial and snap-frozen in liquid nitrogen. The residing CCM was discarded and immediately cells were washed twice with 2 mL of 0.9% of isotonic NaCl solution and quenched with 1 mL ice-cold precipitation solution (acetonitrile/methanol (3:1, *v*:*v*)), containing internal standards (2-Chloroadenosine 100 nM, heptadecanoic acid 1 μg/mL, isoquanosine 100 nM, O-methyl-L-tyrosin 1 μg/mL, phenyl-ß-D-glucopyranoside 1 μg/mL and ribitol 1 μg/mL) [65]. Cells were scraped completely, transferred to a new vial and snap frozen. CCM and cells were stored at −80 °C until analysis.

### 3.4. Preparation of CCM for MS Analysis

While working on ice, 100 µL of CCM was added to 900 µL of ice-cold precipitation solution (acetonitrile/methanol (3:1, *v*:*v*), vortexed and subsequently centrifuged (for 45 min at 20,000× *g* at 4 °C). The supernatant was transferred in 100 µL aliquots for GC analysis and in 300 µL aliquots for LC analysis, and was evaporated until completely dry in a vacuum concentrator. The metabolite pellets for untargeted GC/MS profiling were derivatized as previously described [65], and pellets for targeted LC/MS profiling were reconstituted in 100 µL ultrapure water (18.2 MΩ).

### 3.5. Preparation of Cells for MS Analysis

Cell samples were thawed, vortexed for 5 min at 1400 rpm and subsequently centrifuged for 45 min at 20,000× g at 4 °C. Three aliquots of 300 µL were taken and evaporated to dryness in the vacuum concentrator. One aliquot was derivatized for GC analysis, one was prepared for LC analysis of nucleosides and one for LC analysis of lipids (pellets for lipid analyses were treated under a nitrogen atmosphere).

### 3.6. Untargeted GC/MS Profiling

Dried metabolite pellets were subjected to untargeted GC/MS-based profiling as described previously [66]. In summary, 30 µL of each sample, containing all metabolites, was transferred into GC vials for untargeted analysis via GC electron ionization and single quadrupole MS, and 30 µL from each sample was pooled to create a mixed quality control sample. Features were aligned by SpectConnect [67] and annotated according to retention index and mass spectral similarity to different libraries, including an in-house reference database. This in-house database was established with authentic standards measured on the same system with the same method. This database covers the most important primary metabolites, including amino acids, sugars, sugar phosphates, sugar alcohols, organic acids, polyamines, nucleosides and nucleobases, as well as simple lipids. Metabolite intensities were normalized to phenyl-ß-D-glucopyranoside and peak-sum. Statistical analysis was conducted with MetaboAnalyst 6.0.

### 3.7. Targeted LC/MS Analysis of Nucleosides

The targeted analysis of nucleosides was performed as described previously [65]. In summary, nucleosides were separated by reversed-phase HPLC (Waters Acquity HSS T3, Waters GmbH, Eschborn, Germany; Agilent LC 1290 Infinity, Agilent Technologies, Waldbronn, Germany) coupled to a triple quadrupole mass spectrometer (Agilent Technologies 6460 Triple Quad LC/MS). 70 µL of the aqueous phase, containing nucleosides, was transferred into LC vials for targeted analysis Via HPLC triple quadrupole MS, and 10 µL from each sample was pooled to create a mixed quality control sample.

### 3.8. Targeted LC/MS Analysis of Lipids

The targeted analysis of lipids was performed as described previously [68]. In summary, lipid pellets were dissolved in 100 µL lipid resuspension solution (Isopropanol/ACN/H_2_O 2:1:1, *v*/*v*) kept at 15 °C and subjected to targeted LC/MS lipid profiling. A BEH C18 (2.1 × 100 mm, 1.8 μm) column (Waters Corporation, Milford, MA, USA) was used with the chromatographic program as previously described [69]. 70 µL of the lipid solution was transferred into an LC vial for targeted analysis via HPLC triple quadrupole MS, and 10 µL was pooled to create a mixed quality control sample.

### 3.9. RNA Isolation

Total RNA was extracted from cultured cells using the EURx GeneMATRIX Universal RNA Purification Kit for isolation of total RNA and miRNA from cell culture (Roboklon GmbH, Berlin, Germany/EURx Ltd., Gdansk, Poland) according to the manufacturer’s protocol and as previously described [65]. The RNA concentration of each sample was quantified using the portable UV/Vis spectrophotometer NanoPhotometer™ N60 (Implen GmbH, München, Germany) and stored at −20 °C until use.

### 3.10. Reverse Transcription and Quantiative PCR

For cDNA synthesis, 1 µg of isolated RNA per sample was used for Poly(A) tailing-based reverse transcription (RT). The reaction mixture consisted of 5 µL of 5× RT buffer (in house), 1 µL of reverse transcriptase primer (in house), 1 µL of dNTPs (Jena Bioscience, Jena, Germany), 0.25 µL of Maxima Reverse Transcriptase (Thermo Fisher Scientific, Waltham, MA, USA), 0.25 µL of SUPERase·In RNase inhibitor (Invitrogen, CA, USA) and nuclease-free water to a final volume of 25 µL. The RT reaction was performed in a thermal cycler (Mastercycler, Eppendorf, Hamburg, Germany) under the following conditions: 25 °C for 10 min, 50 °C for 30 min, followed by enzyme inactivation at 85 °C for 10 min. Subsequently, cDNA concentrations were quantified using a NanoPhotometer™ N60 UV/Vis spectrophotometer (Implen GmbH, Munich, Germany). The processed cDNA was stored at 4 °C until further use.

Relative expression levels of specific metabolic enzymes and HIF-1α were determined by quantitative PCR (qPCR). For each sample, 1 µL of cDNA was mixed with 9 µL of qPCR master mix, consisting of 1 µL of 10× qPCR buffer (in house), 0.5 µL of dNTPs (Jena Bioscience, Jena, Germany), 0.5 µL of gene-specific primer (Integrated DNA Technologies, Leuven, Belgium), 0.5 µL of SYBR Green (Roche GmbH, Grenzach-Wyhlen, Germany), 0.05 µL of HotStart Taq Polymerase (Jena Bioscience, Jena, Germany) and 6.45 µL of nuclease-free water. The qPCR was carried out using a LightCycler^®^ 480 II thermal cycler (Roche GmbH, Grenzach-Wyhlen, Germany) under the following cycling conditions: initial denaturation at 95 °C for 5 min, followed by 40 amplification cycles of 95 °C for 10 s, 60 °C for 20 s, and 72 °C for 20 s, with a final extension at 65 °C for 60 s. Data analysis was performed using LightCycler^®^ 480 Software Version 1.5.1.62 (Roche GmbH, Grenzach-Wyhlen, Germany). No-template controls (NTCs) without cDNA were included to confirm the absence of contamination. Relative expression of target genes was normalized to ER membrane protein complex subunit 7 (EMC7) expression and calculated as fold change across all samples using the 2^−ΔΔCt^ method.

### 3.11. Data Processing and Statistical Analysis

The GC-MS data were annotated according to the retention index and mass spectral similarity to different libraries, including an in-house database. The LC-MS data were processed using Agilent MassHunter Qualitative Analysis (version B.07.00) and Agilent MassHunter Quantitative Analysis (version B07.01 SP2). Intensities of lipids, metabolites and nucleosides were normalized to an internal standard, and, by the sum of all peaks, the latter applies only to MS data from cells. Statistical analysis was conducted with Microsoft Excel 2024 and MetaboAnalyst 6.0. Missing values of the metabolomics datasets were replaced by one fifth of the minimum values of each feature and were range-scaled. One-way ANOVA was used for significance testing, followed by Tukey’s honestly significant difference (HSD) test for post hoc analysis. Images, such as heat maps and PCAs, were created with MetaboAnalyst 6.0. The heat maps only show significantly altered lipids, metabolites or nucleosides detected by ANOVA. Euclidean’s distance measure and Ward’s clustering method have been used for heat maps. Significances were shown with the following symbols *: *p* < 0.05, **: *p* < 0.01 and ***: *p* < 0.001

## 4. Conclusions

This study provides novel insights into the metabolic landscape of endometrial cancer (EC) by integrating untargeted GC/MS and targeted LC/MS metabolomic profiling of EC cell lines under standard and stress conditions. For the first time, a metabolic subtyping of EC was proposed based on intracellular and extracellular metabolite patterns, supported by principal component analysis and hierarchical clustering. Two distinct metabolic subtypes of EC cells can be classified: EC subtype 1 (AN3-CA and EFE-184), marked by high biosynthetic activity, elevated hexosyl-ceramide and cholesterol synthesis and metabolites linked to enhanced RNA stability, and EC subtype 2 (HEC-1B and MFE-296), characterized by increased phospholipid metabolism and a predominance of methylated adenosines and guanosines.

**Figure 8:**
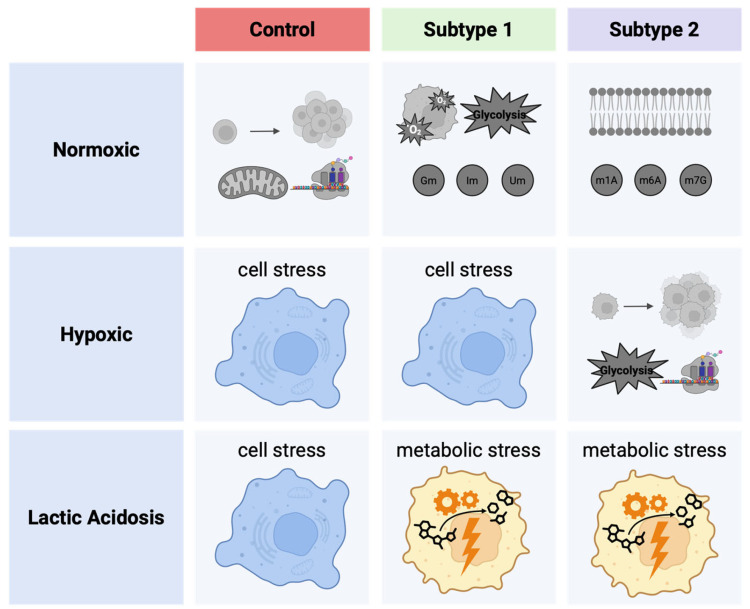
Characteristics of metabolic subtyping of endometrial cancer cell lines. Created in BioRender. Lillemeier, B. (2025). Available online: https://BioRender.com/3zdh95c (accessed on 19 September 2025).

The differential metabolic responses of these subtypes to environmental (often cancer-associated) stressors further reinforce the biological relevance of this classification. Under hypoxia (HX), cells of EC subtype 1 exhibited clear signs of metabolic stress, whereas cells of EC subtype 2 showed adaptive metabolic shifts, including increased amino acid, pyruvate, and lactate levels, indicative of stress resistance. In contrast, both subtypes responded similarly to lactic acidosis, suggesting a convergent survival strategy under acidotic conditions. Importantly, the detection of distinct tumor-associated carbohydrate antigens—glucosylceramides in subtype 1 and lactosylceramides in subtype 2—may offer avenues for personalized therapeutic targeting. Key findings are summarized in Figure 8.

Although the four EC cell lines used in this study were derived from different donors and thus reflect inter-donor heterogeneity, representing four biologically distinct tumors, we were able to identify two distinct metabolic subtypes. This is a particularly interesting finding, as it suggests that despite the underlying molecular and genetic diversity of the individual tumors, certain metabolic programs converge into shared subtype-specific patterns. These results highlight the potential of metabolic profiling as a complementary approach to stratify EC beyond traditional histopathological or molecular classifications.

Collectively, these findings not only confirm known hallmarks of cancer metabolism but also introduce a potentially clinically relevant metabolic stratification of EC. To realize its translational potential, future studies should validate these metabolic subtypes in three-dimensional culture models, where organoids or spheroids can be used as a next step, and ultimately in patient-derived samples, and compare them with current molecular classification systems from the TCGA. Such efforts could bridge the gap between metabolic phenotyping and precision oncology in endometrial cancer.

## Figures and Tables

**Table 1:** Cell lines used in this study. Basic information provided by DepMap Portal [28] and Cellosaurus [29]. GoF = gain of function {XE “Gain of Function” \t “GoF”}; hTERT = human telomerase reverse transcriptase; LoF = loss of function; MSI = microsatellite instability; uk = unknown.

Cell Line	Disease (Celltype)	Donor Age (Years)	Donor Ethnicity	Genetic Alteration
AN3-CA	Endometrial carcinoma	55	Caucasian	LoF: TP53 MSI-high
EFE-184	Endometrial carcinoma	69	Caucasian	LoF: TP53 MSI-stable
HEC-1B	Endometrial carcinoma	71	Asian	GoF: KRAS, PIK3CA, ERBB2 LoF: TP53 MSI-high
MFE-296	Endometrial carcinoma	68	Caucasian	GoF: PIK3CA LoF: TP53 MSI-high
THESC	Healthy	uk	Uk	hTERT-immortalized human endometrial stromal cells (with fibroblast morphology)

## Data Availability

The original contributions presented in this study are included in the article/Supplementary Materials. Further inquiries can be directed to the corresponding authors as highlight of updated file.

## Supplementary Materials

The following supporting information can be downloaded at: https://www.mdpi.com/article/10.3390/ijms26199573/s1.
