# Supplementary material for: Unveiling Metabolic Subtypes in Endometrial Cancer Cell Lines: Insights from Metabolomic Analysis Under Standard and Stress Conditions
Source: Int J Mol Sci. 2025 Sep 30;26(19):9573. doi: 10.3390/ijms26199573 (PMC12524432; doi:10.3390/ijms26199573)
Supplement: Supplementary file 1 [file ijms-26-09573-s001.zip › ijms-3873457 supplement.pdf]

International Journal of *Molecular Sciences*

MDPI

Article

Unveiling Metabolic Subtypes in Endometrial Cancer Cell Lines: Insights from Metabolomic Analysis Under Standard and Stress Conditions - Supplementary

Lana McCaslin[1,4], Simon Lagies [1,2], Daniel A. Mohl [1,2], Dietmar A. Plattner [2], Markus Jäger [4], Claudia Nöthling [4], Matthias C. Huber [4], Ingolf Juhasz-Böss [4], Bernd Kammerer [1,2,5,6*] and Clara Backhaus [4*]

[1] Core Competence Metabolomics, Hilde-Mangold-Haus, University of Freiburg, 79104 Freiburg, Germany; daniel.mohl@ocbc.uni-freiburg.de (D.A.M.)
[2] Institute of Organic Chemistry, University of Freiburg, 79104 Freiburg, Germany
[3] Pharmaceutical Bioinformatics, Institute of Pharmaceutical Sciences, University of Freiburg, 79104 Freiburg, Germany
[4] Department of Obstetrics & Gynecology at the Medical Center-University of Freiburg, Hugstetter Str. 55, 79106, Freiburg, Germany.
[5] Signaling Research Centre BIOSS, University of Freiburg, 79104 Freiburg, Germany
[6] Spemann Graduate School of Biology and Medicine (SGBM), University of Freiburg, 79104 Freiburg, Germany
[*] Correspondence: Bernd Kammerer (BK) and Clara Backhaus (CB) share the correspondence for this article. Contact: bernd.kammerer@ocbc.uni-freiburg.de (BK); clara.backhaus@uniklinik-freiburg.de (CB)

Supplementary Table S1: List of all significant nucleosides with their corresponding abbreviation.

**Abbreviation**	**Nucleoside**
8-OH-G	8-Hydroxyguanosine
A	Adenosine
ac4C	N4-Acetylcytidine
acp3U	3-(3-Amino-3-Carboxypropyl)Uridine
AICAR	5-Aminoimidazole-4-Carboxamide Ribonucleotide
C	Cytidine
Cm	2'-O-Methylcytidine
D	Dihydrouridine
G	Guanosine
Gm	2'-O-Methylguanosine
I	Inosine
i6A	N6-Isopentenyladenosine
Im	2'-O-Methylinosine
m1A	1-Methyladenosine
m1G	1-Methylguanosine
m1I	1-Methylinosine
m1Y	1-Methylpseudouridine
m2,2,7G	N2,N2,7-Trimethylguanosine
m2,2G	N2,N2-Dimethylguanosine
m2G	N2-Methylguanosine
m3C	3-Methylcytidine
m5C	5-Methylcytidine

m5U	5-Methyluridine
m6,6A	N6,N6-Dimethyladenosine
m6A	N6-Methyladenosine
m6Am	N6,2'-O-Dimethyladenosine
m7G	7-Methylguanosine
ms2i6A	2-Methylthio-N6-Isopentenyladenosine
MTA	5′-Methylthioadenosine
N6SAR	N6-Succinyl Adenosine
P	Pseudouridine
SAH	S-Adenosylhomocysteine
SAM	S-Adenosylmethionine
t6A	N6-Threonylcarbamoyladenosine
U	Uridine
Um	2'-O-Methyluridine
X	Xanthosine

Supplementary Table S2: List of all abbreviated lipids.

**Abbreviation**	**Lipid**
DHCeramide	Dihydroceramide
FFA	Free fatty acid
Hex-ceramide	Hexosylceramid
Lac-cer	Lactosylceramid
PC	Phosphatidylcholine
PC	Phosphatidylethanolamine
SM	Sphingomyelin
TG	Triglyceride

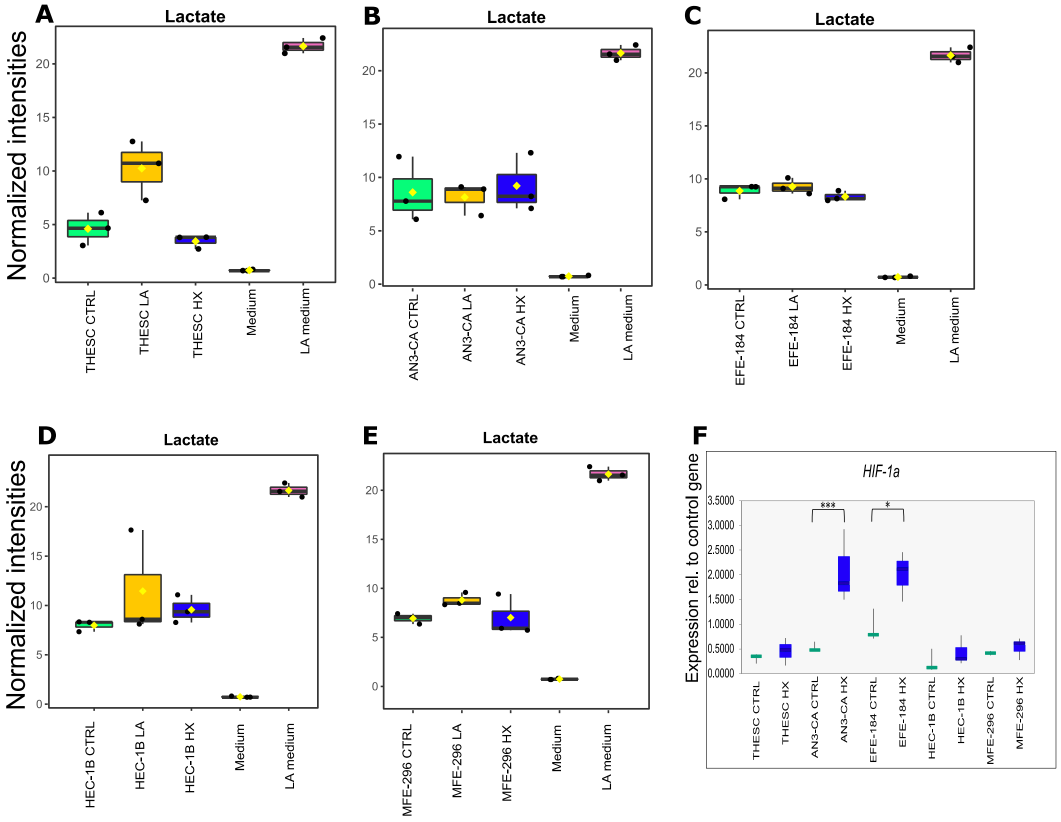

*Supplementary Figure S1: Validation of acidosis and hypoxia treatment. Extracellular lactate concentration (A) – (E) and RNA expression of endogenous hypoxia marker HIF-1α in EC cells (F). (A) – (E) Box plot showing the expression of lactate in three conditions (CTRL = control, AC = lactic acidosis, HX = hypoxia) in respective CCM of THESC cells (A), AN3-CA cells (B), EFE-184 cells (C), HEC-1B cells (D) and MFE-296 cells (E). Normalized intensities to internal standards. Each cell line was analyzed in biological triplicates (N = 3). (F) RNA expression was analyzed for Hypoxia inducible factor-1α (HIF-1α). Expression of ER Membrane Protein Complex Subunit 7 (EMC7) served as a control gene. Significances were shown with symbols *: $p < 0.05$, **: $p < 0.01$, ***: $p < 0.001$. Each cell line was analyzed in biological triplicates (N = 3).*
